# Supplementary material for: Central catheter tip migration in critically ill patients
Source: PLoS One. 2022 Dec 19;17(12):e0277618. doi: 10.1371/journal.pone.0277618 (PMC9762564; doi:10.1371/journal.pone.0277618)
Supplement: Attachment — Submitted filename: Response to Reviewers.docx [file pone.0277618.s002.docx]

**Dear Martin Kieninger,**

Academic Editor
PLOS ONE

We are pleased to submit the revised version of the manuscript entitled " **Central catheter tip migration in critically ill patients**" (formerly named *Assessing Central Venous Catheter tip location- a different approach?*) [PONE-D-22-14765]

We greatly appreciate the efforts and extensive and constructive comments of the referees. A point-by-point response follows, and description of all changes to the manuscript has been submitted (the changes are highlighted in the manuscript).

It is our belief the manuscript is improved as the result of the reviewers’ thoughtful and constructive comments and suggestions for revision.

Reviewer #1,

**thank you for submitting your article and data. I believe that the article needs some major revision to clarify the message for the readers. I hope that my suggestions help you to improve your article.**

Major concerns:

**1. The title of your work suggests that you present a new/different approach to controlling catheter location with x-rays. But, you do not! Instead, you investigate catheter migration over time with consecutive x-rays. Choose an appropriate title.
In the discussion section, you write: …examine the movement of CVC tip throughout its use. Exactly, this should be reflected in the title.**

Thank you for this important comment. We have changed the title of the article to better represent our study and conclusions. We have changed the title to " Central catheter tip migration in critically ill patients". We feel this title is appropriate for our study.

2) **Follow up is 72 hours? Why? You state that every x-ray was evaluated in the method section. Why did you only use a 72 hour follow up as mentioned in the abstract and figure 3 and 4?**

The decision to evaluate the first days of hospitalization was because we wanted to focus on the early phases of the ICU admission for several reasons – first, our interest was about the technique of confirmation of CVC tip location and how it may be influenced by the documentation of CVC tip migration, second, after several days we anticipated that the frequency of X rays of each and every patient would decrease and so our data could become less accurate. Third, other factors could have had different influence on CVC tip location– routine patient movement and position changes, patient extubation, using armchair, etc. although all of these are legitimate questions, we felt that they are beyond the scope of our question.

Regarding 72h- we did not evaluate only the first 72 hours but evaluated every chest x-ray taken. We used only 4 x-rays for each patient (initial after insertion and 3 follow up x-rays) for the reason stated above. The study was not limited to 72 hours- for most patients the third follow up was taken between 72 to 96 hours from CVC insertion. Thank you for clarifying this, we changed the results and discussion sections to better explain this accordingly.

3) Material and Method
**Clarify, how patients were included into the study. Do you have a database. Did you screen your PDMS, etc. for every patient admitted between xxx and xxx?**

We apologize for the lack of clarity and thank you for pointing it out. We screened our EMR (electronic medical records) database for every patient admitted to ICU or CVICU between January to June 2019. We selected only patients with CVC and at least 2 follow up CXR's during hospitalization (taken before CVC extraction/other manipulation to the CVC). We excluded patients as mentioned in the exclusion criteria. Then we selected only patients with CVC inserted to the right internal jugular in order to minimize the effect of CVC location on our results. We changed the method section to better explain this point.

**4) The exclusion criteria are documented sufficiently.**

**5) How was the mentioned distance between carina and catheter tip measured? Did you use a software? Did you use a x-ray fluoroscope/screen? A difference between tow investigators of more than 1 cm is mentioned – this seems large using a software?**

We used "Vue Motion", PHILIPS, Version12 software for picture archiving and communication, that allows enlargement and contrast enhancement for a clearer view and measurement of the anatomical and catheters positions. The distance was then measured using distance measuring tool imbedded in the software- the same tool routinely used in the ICU for x-ray evaluation. As some X rays are of poor quality (taken in the ICU using portable machine), the exact location of the Carina was sometimes difficult to find- We believe this was the reason for the difference.

**6) Who are the investigators? Part of your study team or part of the wards? What was there training? How did the training of the ICU specialist differ how was consulted in case of poor x-ray quality? Did you check with radiology?**

We did not routinely consult a radiologist as the practice in our institute is that the intensivist caring for the patient is reviewing the X rays immediately post CVC insertion and throughout its use. radiologists are consulted whenever the attending physician is not sure about the exact location or a specific concern exists, otherwise, radiologist will review the X ray in a later time and so most of the CVC repositioning are being made according to the intensivist interpretation of the imaging. We kept the study as practiced in everyday practice in our institute.

Our investigators were Dr. Merin and Dr. Vine- part of the study team, both are physicians working in the ICU and examining CXR as part of daily practice. Initial training with a radiology physician was conducted to select the method the location of CVC tip should be measured compared to the carina. In any case of discrepancy of over 1 cm, Dr. Stavi (an ICU specialist with over 15 years of experience in the ICU) examined the CXR to determine the position.

**7) Statistics. You used a T-test. Did you check for normal distribution? If data does not show normal distribution a non-parametric test should be used.**

We checked for normal distribution of the data prior to preforming the T-test. Thank you for pointing this issue, an appropriate explanation was added to the method and results sections accordingly.
 **8) The multi-parameters regression model used in table 3 has to be explained in the method section.**

Thank you for the comment. The multivariant analysis was performed using linear regression as the main outcome variable is catheter tip migration. A comment was added to the method section.

Results and Discussion

**9) Major influencing factors of possible catheter mobilizations are not investigated or mentioned; e.g. prone positioning, mobilization of patients, insertion duration of the catheters, etc. If these data are not available, it should at least be discussed as limitation**.

Agreed – none of the study patients needed prone position during the study. As for mobilization – it is routine to change position of the patients every several hours- we documented the patient's position while the x-ray was taken (supine or sitting). We excluded patients who underwent major mobilization (such as changing of hospital wards or surgery) during the study. As of insertion duration- part of the study was measuring CVC movement over time, as shown in figure 3+4. Other factors effecting catheter mobilization, as head position and anatomical variance are mentioned in the limitations section

**10) Adverse events/e.g. the non-existence of adverse events is not mentioned or discussed. Not one catheter had to be removed (even the one in the subclavian vein)?**

No adverse events were documented. since CVC complications are not frequent, we estimated that in order to evaluate safety a much larger population in needed.

As for the CVC that went into the subclavian, we are aware that in other cases, the CVC should have been removed, but in this case, the responsible physician decided to keep it as he was not interested in measuring central venous pressures or gases. It was also estimated that the disadvantages of removing the current CVC and inserting a new one in that specific patient could be more significant than the advantages. in our perspective this emphasises the diversity between different physicians as for tolerating different CVC tip positions.

We added a comment to the text to better reflect the issue regarding adverse events during the study.

Conclusions

**11) You only included 101 patients. Relativize your conclusion: ….. a 15 cm CVC inserted through the RIJ in most adults will likely initially reside ….. Refer to the height and weight of the studied patients.**

Thank you for pointing this out. This is indeed a major part of our conclusions. A comment regarding the height and weight of those patients was added to the conclusions section.

General
**I suggest to revise the paper. Focus on your major statement: catheters may move by several centimeters of the course of time (with or without major adverse events???).

Include data to these observed/non-observed adverse events? After all, what does the catheter movement mean for our patients/clinical practice?**

We thank you for the meaningful comments and suggestion regarding our study. The goal of the study was to describe CVC movement during first days of hospitalization- as many publications deal with correct CVC positioning, but whether initial positioning has any effect on future CVC positioning was yet to be described. Regarding adverse effect- CVC adverse effects are usually rare (according to past publications) and are evaluated less then 1%; for this reason, our study goal was not to examine CVC complications (a much larger population is needed for that). We believe that the combination of low complications rate and CVC tip movement in and out from a correct (narrow) position (as we describe in our study), raises questions regarding the routine practice of initial CVC tip position examination.

Minor concerns

Abstract:
**12) Design and Settings: Retrospective, single-center study, conducted in the Intensive Care and Cardiovascular Intensive Care Units between January and June 2019….
- State the name of the hospital/institution here including city and country**

Accepted and changed accordingly

**13) The average initial tip position was 1.52 (±1.9)…..

- Clarify, you probably refer to mean ± SD**

Yes, we mean SD. Thank you for the comment, Accepted and changed accordingly. Comment added also to the result section of the article.

**14) Central Venous Catheterization (CVC) is a common procedure in intensive care units (ICU), operating rooms (OR), and other hospital departments.**

**- Specify ‘other hospital departments’ or give examples ‘other departments like…..’ or delete.**

Accepted and changed accordingly- additional examples of department were added.

**15)…a routine chest XRay (CXR) is obtained after insertion and before using the CVC in order to detect insertion related complications and to assess catheter tip position (CTP).

- Really? One x-ray after insertion and a new one before use? How long do you wait until you use newly inserted CVCs in your ICU?**

In our facility we do only one CXR after insertion, using the CVC is allowed only afterwards. In the ICU we usually wait up to an hour or two for this CXR, while in other wards it might take even longer. We have rephrased the sentence to explain this better.

**16) In addition, the use of bedside-US has been shown to be faster and more accurate in detecting these complications when they occur.

- Specify, faster than what else (x-rays)?**

Yes, we mean faster the CXR. Changed accordingly

17) **CTP- You already use CVC as abbreviation. I suggest to use only CVC and refer to the ‘tip of the CVC’, since both abbreviations are similar.**

Accepted, changed accordingly throughout the text.

**18)….performing US assisted or guided CVC insertion. Clarify. What is the difference?**

We referred to the article published by Bernd Saugel and Jean-Louis Teboul: Ultrasound-guided central venous catheter placement: a structured review and recommendations for clinical practice (reference 3), where US assisted means "Static" US (also called indirect US), a technique using US only before CVC placement to identify the anatomy of the target vein, while US guided means "real-time" US- a technique of needle advancement and vessel puncture under permanent US control.

We believe this issue has only a small effect on the study, so we referred only to US-assisted.

**19) …m the upper part of the SVC to the right atrium10,13–19 (Zone A and B).

- Clarify, Zone A and B as** **depicted in figure xxx.**

Accepted, changed accordingly.

**20) All data were analysed by SPSS version 21.0.

- Suggestion: All data were analysed using SPSS version 21.0.**

Accepted, changed accordingly.

**21) At each follow-up CXR, the chance of an initially well-positioned and initially malpositioned catheter to be in the "safezone" were similar (48% vs 39% at first CXR, 49% vs 26% at second and 40% vs 50% at third CXR.**

**- Do not use ‘safezone’, use the terminology introduced above. You refer to similar, but I do not see a statistical evaluation. If a descriptive approach was used it has to be explained in the method section.**

Regarding the 'Safe Zone terminology'- accepted and changed accordingly.

Regarding the statistical evaluation: a descriptive approach was selected due to the relatively small sample size. We believe that even if there is any statistical difference between the groups, the main issue is that at every follow up, regardless of initial position, a CVC has a high likelihood (between 50-60% according to our study) of ending malpositioned. A comment regarding the descriptive approach was added to the method section accordingly.

**22) We examined both the range of daily movement…

- No! Do you perform routine daily x-rays on your ICU? If so, explain in the method section. This would contribute to the work. Explain, when follow up x-rays were taken.**

In the first days after CVC insertion the patient may be unstable, and for this reason the clinical team request a CXR at a high frequency (our study was retrospective and did not change in any way the clinical decisions to perform chest x-rays). Once the patient is stabilized the time between CXR's is usually longer and is decided according to the physician's decision. In almost all selected cases, all 4 CXR's (initial and the three follow up CXRs) were held between 72-96 hours, maximum time from CVC insertion to last CXR was 5 days.

Thank you for the correction and we apologize for the lack of accuracy. We have changed the text to better clarify this. We also applied this change to figures 3+4 (scale of initial, 2^nd^ 3^rd^ and 4^th^ CXR's, not 24,48,72 hours)

**23) There was a significant average movement of was 1.9 (±1) cm of CTP and thus predicting accurately the tip position (within a narrow range) is limited.

- Does not make sense. Clarify. Of course, one cannot predict a future positioning because of a current exact position.**

Accepted, the text was not written in a correct matter. Changed accordingly.

**Reviewer #2:**

This manuscript is a retrospective study on assessing the migration of the tip of IVC in hospitalized patients. The authors have concluded that the tip of CVC can migrate throughout patients’ hospitalization course, but “will remain within a wide range of the top of the right atrium and the middle of the Superior Vena Cava (SVC), if accepted as well-positioned”.

In terms of the originality of the article, there have been descriptions of several cases of catheter migration, but I could not find any prospective or retrospective studies in this area.

1) **The title of this manuscript could be changed, it should be more explanatory of the study itself. Please, consider changing the title. The abstract portion of the manuscript clearly reflects its content.**

Thank you for this important comment. We have changed the title of the article to better represent our study and conclusions. We have changed the title to " Central catheter tip migration in critically ill patients". We feel this title is appropriate for our study.

**2) In results in Table 2, please specify the descriptions. If catheter was malpositioned since initial placement, how you can make a column of “Well-positioned catheter”. Consider revising.**

We apologize for the lack of clarity. In table 2 we present the likelihood of the CVC to be wellpositioned or malpoistioned in CXR's during hospitalizations, based on the initial position of the CVC. The top part shows the 46 initially well positioned CVC and their follow up while the second part shows initially mal positioned. Some of initially malpositioned CVC migrated during hospitalization into the well-positioned area and vice versa.

We have changed the table and the text preceding it to better explain this.

**3) In the discussion, you should also add several limitations of the study secondary to its retrospective origin:
1. Even though catheters were placed by two teams of providers, there is a necessity to mention that difference in providers skills should be a consideration**

In our facility, in the ICU and CVICU all catheters are inserted by ICU or anesthesia physicians- both undergoing training at the same program. We agree that each have his own skill and hence could be a difference between providers. We changed the text to better explain this point.

**2. There was no mention of how the CVCs were secured in place. That also might add to a higher or lower possibility of migration, depending on how CVCs were secured and dressings applied.**

Thank you for the comment, we agree that securing the CVC can affect movement. In our facility all CVC's are inserted according to institutional directives which are taken from the requirements of the ministry of health- this means stitching the CVC to the skin and properly dressing the CVC.

We added the method in which the CVC was secured and dressed to the results sections.

**In conclusion, I would recommend this Manuscript for publication with the change of the title and minor correction as I mentioned above**.
