# Supplementary material for: Central catheter tip migration in critically ill patients
Source: PLoS One. 2022 Dec 19;17(12):e0277618. doi: 10.1371/journal.pone.0277618 (PMC9762564; doi:10.1371/journal.pone.0277618)
Supplement: Attachment — Submitted filename: Response to Reviewers.docx [file pone.0277618.s003.docx]

**Dear Martin Kieninger,**

Academic Editor
PLOS ONE

We are pleased to submit the revised version of the manuscript entitled " **Central catheter tip migration in critically ill patients**" [PONE-D-22-14765]

We greatly appreciate the extensive efforts and constructive comments of the referees. A point-by-point response follows, and a description of all manuscript changes has been submitted (the changes are highlighted in the manuscript).

We believe that the manuscript has improved due to the reviewers’ thoughtful and constructive comments and suggestions for revision.

Reviewer #1:

1) **Another limitation rests with the retrospective origin of the trial- CVC's were inserted by different physicians, and although all were ICU or Anaesthesia physicians with similar training working under the same guidelines, of nature each have different skill that were not taken into account.
-> Please, consider to break it down.**

We apologize for the lack of clarity. The sentence was rephrased.

**2) Some typos 'sneaked' into the revised version:

... with hight over 155 cm and BMI<40....

-> Should be: height**

**The article needs to be checked for typos and akward phrasing before publication.**

Thank you for the important comment. We have revised the article again for any spelling or grammar errors.

Reviewer #2: Please make minor changes to the manuscript:

1) **Materials and methods: -"Patients under the age of 18, hospitalized less than 24 hours, prone position, pregnant individuals..." - Add ‘Patients in' prone position...**

Thank you for the comment, the sentence was corrected accordingly.

2)**-“****After proving normality“ - please revise**

Correct- we changed to "Data was proved for normal distribution prior to performing T-test". Thank you for pointing out this issue.

**3) Table 2: when describe the “Malpositioned CVCs” you have to change the name of “Well-positioned tip”, since there is no well-positioned tip in mal-positioned catheters. You possibly mean that the catheter tip was not migrated from initial malposition, but it is not well understood from the description - revise.**

We apologize for the lack of clarity. The table shows the migration of catheter tips- in the first part, we follow the 46 catheters that were initially well-positioned and indicated whether the tip remained well-position or migrated to malposition in following CXR's. The second part of the table relates to catheter tips that were initially mal-positioned and indicate their migration to the well-position zone.

We have revised the headlines to better indicate this properly.

We would like to thank you again for these important comments.
